# Use of Viremia to Evaluate the Baseline Case Fatality Ratio of Ebola Virus Disease and Inform Treatment Studies: A Retrospective Cohort Study

**DOI:** 10.1371/journal.pmed.1001908

**Published:** 2015-12-01

**Authors:** Oumar Faye, Alessio Andronico, Ousmane Faye, Henrik Salje, Pierre-Yves Boëlle, N’Faly Magassouba, Elhadj Ibrahima Bah, Lamine Koivogui, Boubacar Diallo, Alpha Amadou Diallo, Sakoba Keita, Mandy Kader Konde, Robert Fowler, Gamou Fall, Simon Cauchemez, Amadou Alpha Sall

**Affiliations:** 1 Arbovirus and Viral Hemorrhagic Fever Unit, Institut Pasteur de Dakar, Dakar, Senegal; 2 Mathematical Modelling of Infectious Diseases Unit, Institut Pasteur, Paris, France; 3 Department of Epidemiology, Johns Hopkins Bloomberg School of Public Health, Baltimore, Maryland, United States of America; 4 INSERM, UMR-S 1136, Institut Pierre Louis d’Epidémiologie et de Santé Publique, Paris, France; 5 Sorbonne Universités, UPMC Univ Paris 06, UMR-S 1136, Institut Pierre Louis d’Epidémiologie et de Santé Publique, Paris, France; 6 Laboratoire de Fièvres Hémorragiques de Guinée, Hôpital Donka, Conakry, Guinea; 7 Service des Maladies Infectieuses, Médecins Sans Frontières, Conakry, Guinea; 8 National Public Health Institute, Conakry, Guinea; 9 World Health Organization, Conakry, Guinea; 10 Ministry of Health, Conakry, Guinea; 11 Centre d’Excellence de Formation & Recherche sur le Paludisme & les Maladies Prioritaires en Guinée, Conakry, Guinea; 12 University of Toronto, Toronto, Ontario, Canada; Mahidol-Oxford Tropical Medicine Research Unit, THAILAND

## Abstract

**Background:**

The case fatality ratio (CFR) of Ebola virus disease (EVD) can vary over time and space for reasons that are not fully understood. This makes it difficult to define the baseline CFRs needed to evaluate treatments in the absence of randomized controls. Here, we investigate whether viremia in EVD patients may be used to evaluate baseline EVD CFRs.

**Methods and Findings:**

We analyzed the laboratory and epidemiological records of patients with EVD confirmed by reverse transcription PCR hospitalized in the Conakry area, Guinea, between 1 March 2014 and 28 February 2015. We used viremia and other variables to model the CFR. Data for 699 EVD patients were analyzed. In the week following symptom onset, mean viremia remained stable, and the CFR increased with viremia, *V*, from 21% (95% CI 16%–27%) for low viremia (*V* < 10^4.4^ copies/ml) to 53% (95% CI 44%–61%) for intermediate viremia (10^4.4^ ≤ *V* < 10^5.2^ copies/ml) and 81% (95% CI 75%–87%) for high viremia (*V* ≥ 10^5.2^ copies/ml). Compared to adults (15–44 y old [y.o.]), the CFR was larger in young children (0–4 y.o.) (odds ratio [OR]: 2.44; 95% CI 1.02–5.86) and older adults (≥45 y.o.) (OR: 2.84; 95% CI 1.81–4.46) but lower in children (5–14 y.o.) (OR: 0.46; 95% CI 0.24–0.86). An order of magnitude increase in mean viremia in cases after July 2014 compared to those before coincided with a 14% increase in the CFR. Our findings come from a large hospital-based study in Conakry and may not be generalizable to settings with different case profiles, such as with individuals who never sought care.

**Conclusions:**

Viremia in EVD patients was a strong predictor of death that partly explained variations in CFR in the study population. This study provides baseline CFRs by viremia group, which allow appropriate adjustment when estimating efficacy in treatment studies. In randomized controlled trials, stratifying analysis on viremia groups could reduce sample size requirements by 25%. We hypothesize that monitoring the viremia of hospitalized patients may inform the ability of surveillance systems to detect EVD patients from the different severity strata.

## Abstract 2

In a retrospective cohort study, Simon Cauchemez and colleagues find viral load can predict case fatality ratios among patients with Ebola Virus.

## Editors' Summary

**Background:**

During the current outbreak of Ebola virus disease (EVD) in West Africa, which started in December 2013, there have been more than 28,000 confirmed, probable, and suspected cases of EVD and more than 11,000 deaths from the disease. Ebola virus is transmitted to people from wild animals and spreads in human populations through direct contact with the bodily fluids (including blood, saliva, and urine) or organs of infected people or through contact with bedding and other materials contaminated with bodily fluids. The symptoms of EVD, which start 2–21 days after infection, include fever, headache, vomiting, diarrhea, and internal and external bleeding. Infected individuals are not infectious until they develop symptoms but remain infectious as long as their bodily fluids contain virus. There is no proven treatment or vaccine for EVD, although several treatments are now being assessed in people following promising laboratory studies. Supportive care—given under strict isolation conditions to prevent the spread of the disease to other patients or to healthcare workers—improves survival.

**Why Was This Study Done?:**

Ideally, the efficacy of a potential treatment for any disease is assessed in a randomized controlled trial, a study that compares outcomes among people chosen at random to receive the treatment with outcomes among people given a placebo (dummy treatment). However, because EVD is frequently fatal, randomized controlled trials of potential treatments are considered unethical. Instead, studies evaluating treatments for EVD usually compare the case fatality ratio (CFR; the number of deaths caused by a disease divided by the number of cases of that disease; a CFR of 100% indicates that everyone who develops the disease dies) among treated patients with a baseline CFR estimated from historical data. But the CFR of EVD varies markedly over time and space for poorly understood reasons (for example, changes in patient care or variations in the detection of people with disease of different severity might change the CFR). Thus, the CFR in the treatment group could differ from the baseline CFR for reasons that are independent of the treatment. To find a way around this problem, in this retrospective cohort study, the researchers investigate whether there is a relationship between viremia (the amount of virus in the blood) and the CFR among patients with EVD.

**What Did the Researchers Do and Find?**

The researchers used laboratory and epidemiological data (for example, patient age and date of symptom onset and death; epidemiology is the study of disease patterns in populations) to investigate the relationship between viremia and CFR among 699 patients with confirmed EVD hospitalized in the Conakry area of Guinea between March 2014 and February 2015. In the week following symptom onset, mean (average) viremia remained stable, and the CFR among the patients increased with the level of viremia. Thus, the CFRs for patients with low, intermediate, and high viremia (defined by the number of virus copies per milliliter of blood) were 21%, 53%, and 81%, respectively. Compared to adults aged 15–44 years, young children (aged less than five years) and older adults had a higher CFR, but children aged 5–14 years had a lower CFR. Notably, the CFR in the study population was 14% higher after July 2014 than in the months of March–July 2014, an increase that coincided with a ten-fold increase in the average level of viremia in the population.

**What Do These Findings Mean?:**

These findings suggest that viremia is a strong predictor of death that can partly explain variations in the CFR of EVD. Because these findings are based on data collected from hospitalized patients, they may not be generalizable to other settings. Importantly, however, these findings provide estimates of CFR by viremia group that can now be used to adjust risk when undertaking clinical evaluations of EVD-specific treatments. That is, by allowing for differing levels of viremia, it will be possible to assess the efficacy of treatments for EVD more accurately in nonrandomized clinical trials. Moreover, the researchers calculate that stratification of patients by viremia group could reduce the sample size needed in any randomized trials that are undertaken (for example, comparisons of two potential treatments) by 25%. Finally, the researchers suggest that monitoring viremia among patients hospitalized for EVD might provide information about the ability of different surveillance systems to detect patients with different levels of disease severity (probability of death).

**Additional Information:**

This list of resources contains links that can be accessed when viewing the PDF on a device or via the online version of the article at http://dx.doi.org/10.1371/journal.pmed.1001908.

- The World Health Organization (WHO) provides information about EVD, information about potential EVD therapies, and regular updates on the current EVD epidemic; a summary of the discussion of a WHO Ethics Working Group Meeting on the ethical issues related trials of EVD treatments is available; the WHO website also provides information about efforts to control Ebola in the field and personal stories from people who have survived EVD
- The UK National Health Service Choices website provides detailed information on EVD
- The US Centers for Disease Control and Prevention also provides information about http://www.cdc.gov/vhf/ebola/EVD

## Introduction

An epidemic of Ebola virus disease (EVD) of unprecedented magnitude has been ongoing in West Africa since December 2013 [1]. As of 23 September 2015, 28,295 confirmed, probable, and suspected EVD cases and 11,295 deaths have been reported to the World Health Organization [2].

Currently, most treatments used in Ebola treatment centers (ETCs) rely on supportive care, but several experimental therapies are being assessed for EVD following promising in vitro and limited in vivo findings [3–5]. The magnitude of the humanitarian and health crisis and high case fatality ratio (CFR) have led to debate on optimal methodologies for evaluating potential treatments [6–11]. While randomized controlled trials (RCTs) generally provide the most solid evidence on efficacy [6–9], randomization to active treatment or placebo has been considered unethical by some when any preclinical or clinical data suggest a treatment effect [10,11]. In the absence of a concurrent randomly allocated control group, Sissoko et al. [4] and Adebamowo et al. [10] recommended comparing the CFR in treated patients to the baseline CFR of patients who did not receive the treatment, with the baseline value being estimated from historical data collected during the current epidemic or during past EVD outbreaks.

Of course, an important limitation of using historical controls is that, in the absence of randomization, the CFR in the treatment group could differ from the baseline CFR for reasons independent of treatment. Indeed, the CFR of Ebola has varied historically, over time and space [1,12,13]. Factors that might drive these variations include patient care, the ability of surveillance systems to detect EVD patients from the different severity strata, time to hospitalization, and strain pathogenicity. Whatever the underlying causes, these variations make it difficult to define baseline CFRs and properly adjust for differences between the study population and historical controls. Irrespective of the study design, there are also important concerns about the statistical power of any treatment study, since recruitment of cases may become difficult in the declining epidemic.

Here, we investigate the relationship between patients’ viremia (viral load) and their probability of death, and we assess how this relationship may be used to explain temporal trends in reported CFRs and inform different treatment study designs.

## Methods

**Fig 1:**
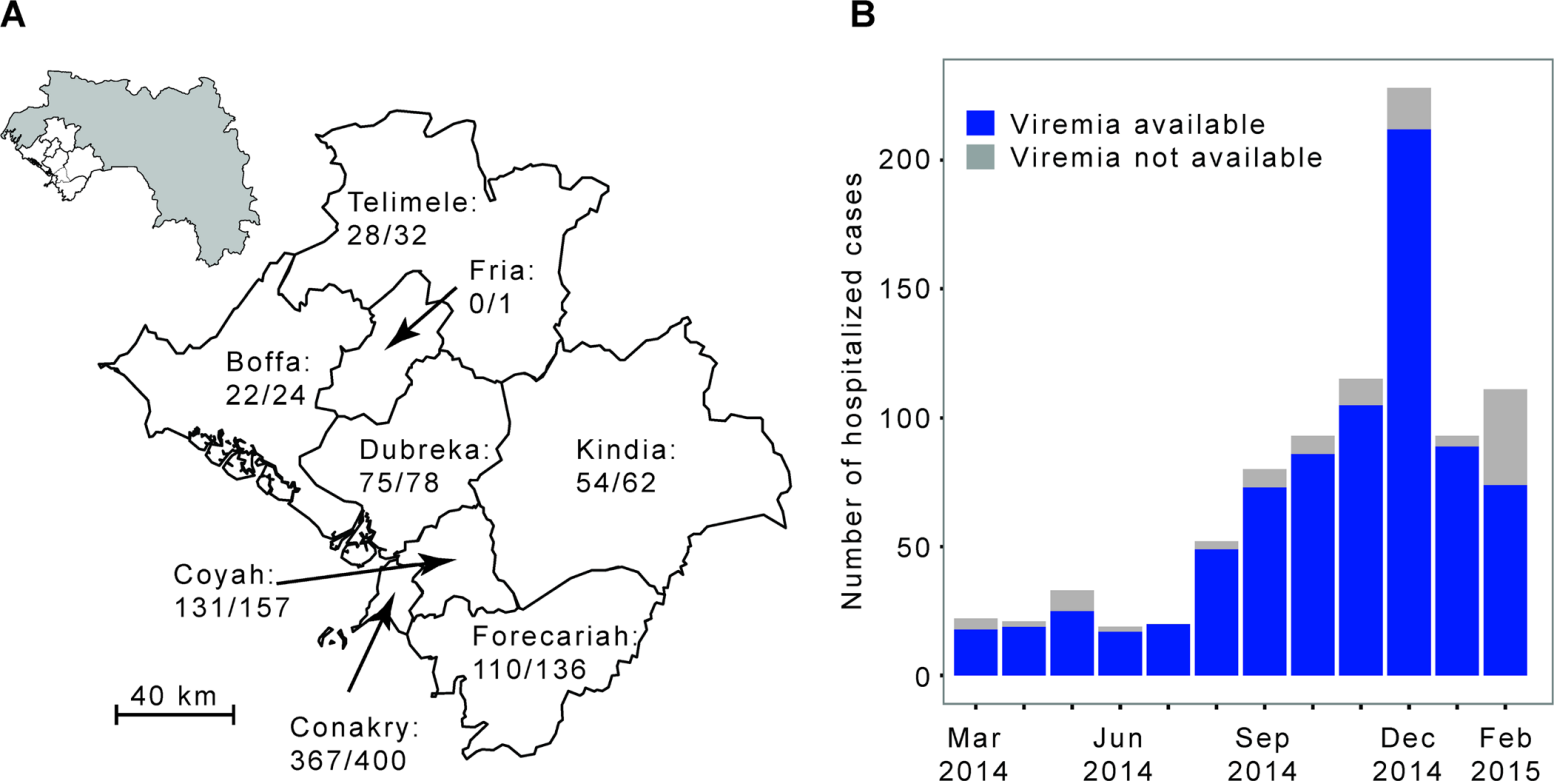
The Ebola virus disease epidemic in the Conakry area, Guinea, March 2014 to February 2015. (A) Map of the study area, which consists of Conakry and the surrounding prefectures of Boffa, Coyah, Dubreka, Forecariah, Fria, Kindia, and Telimele (for which diagnoses were mostly performed by the IPD-LFHP laboratory) (the administrative boundaries were taken from the GADM database; http://www.gadm.org/). (B) Number of cases by month of symptom onset. The total number of probable and confirmed cases in the study area that were hospitalized is indicated in grey. The number of those that were diagnosed by reverse transcription PCR (RT-PCR) by the IPD-LFHP laboratory is in blue.

We analyzed the laboratory results of a large number of EVD patients from Conakry and surrounding prefectures (Fig 1). These patients were tested by the laboratory run by the Institut Pasteur de Dakar (IPD) and the Laboratoire des Fièvres Hémorragiques de Guinée (LFHP) in Conakry.

### Ethical Considerations

We did not seek institutional review board approval for data collection in this study because data were collected as part of routine case management under an emergency response mandate from the government of Guinea. As part of routine practice, patients orally agreed to be tested for Ebola virus infection.

### Laboratory Work

In the IPD-LFHP laboratory in Conakry, diagnosis of EVD was performed using real-time RT-PCR [14,15]. The algorithm for testing is as follows. A blood sample is collected from all suspected EVD patients, and an RT-PCR test is performed systematically. If the test is positive, the case is confirmed. Otherwise, the decision tree for subsequent testing depends on the time *d* from symptom onset to sample collection: (i) if 3 ≤ *d* ≤ 10 d, the negative result is definitive; (ii) if *d* ≤ 2 d, a second sample is collected 3 d after the first sample, and a new RT-PCR test performed, with the results of this second test being final; (iii) if *d* > 10 d, cases are confirmed using a serological test [14,15]. In this paper, analyses were restricted to cases confirmed by RT-PCR for whom viremia was available. Viremia was derived from the *C*_t_ value obtained for each sample tested. We had access to the laboratory dataset, where all the laboratory results were recorded, as available on 3 March 2015.

### Epidemiological Data

The epidemiological line list has already been described in detail elsewhere [1]. In short, a standard case investigation form was used to collect clinical and demographic data for all confirmed, probable, and suspected EVD cases identified through contact tracing and clinical care in Guinea. The following variables were used for our analyses: age, gender, EVD status (confirmed/probable/suspected), prefecture of residence, date of symptom onset, outcome (dead/discharged alive), hospitalization status (hospitalized/not hospitalized), date of hospitalization (if any), date of death (if any), and date of sample collection.

### Inclusion in the Study

Our analysis was restricted to EVD patients from the epidemiological line list who (i) had EVD confirmed by an RT-PCR test performed by the IPD-LFHP laboratory in Conakry, (ii) resided in Conakry or in the surrounding prefectures of Boffa, Coyah, Dubreka, Forecariah, Fria, Kindia, or Telimele (in which diagnoses were mostly performed by the IPD-LFHP laboratory), (iii) had symptom onset between 1 March 2014 and 28 February 2015, and (iv) were hospitalized. Patients were excluded from the analysis if (i) sample collection was done after the day of death or more than 30 d after symptom onset or (ii) one of the following variables was missing or unclear: age, prefecture of residence, date of symptom onset, outcome, date of hospitalization, date of death if died, or date of sample collection. In a sensitivity analysis, we explored the robustness of our findings when different eligibility criteria were used (see Section 4 of S1 Appendix).

### Statistical Analysis

We calculated mean viremia (as measured on a log_10_ scale) as a function of age, gender, and time from symptom onset to sample collection.

We modeled outcome (dead/discharged alive) as a function of viremia *V* in those diagnosed within 1 wk of symptom onset. A univariable logistic regression model with polynomial terms in log_10_ *V* up to degree 5 was used, with best fit selection according to the Akaike information criterion. We also discretized *V* into three groups (low: *V* < *v*_*1*_, intermediate: *v*_*1*_ ≤ *V* < *v*_*2*_ and high: *V* ≥ *v*_*2*_) and selected the best threshold values *v*_*1*_ and *v*_*2*_ (*v*_*1*_ < *v*_*2*_) at maximum likelihood (see Section 2 of S1 Appendix for more details).

We then modeled outcome in all patients in a multivariable logistic regression that included viremia (using the best polynomial term identified above) and the following other predictors of death: age in four classes (young children: 0–4 y.o., children: 5–14 y.o., adults: 15–44 y.o., older adults: ≥45 y.o.) and time from symptom onset to sample collection in three classes (<4, 4–7, >7 d).

Using this model, it was possible to investigate whether the observed temporal variations in CFR could be explained by changes in the distribution of viremia over time. For each case, we computed the probability of death according to viremia, patient age, and time from symptom onset to sample collection. We then averaged these probabilities by month and compared these with the observed monthly CFRs. We explored the consistency of our results when only a subset of cases was used to inform the model. We estimated monthly CFRs using a model that was trained on cases that were hospitalized between 1 March 2014 and 30 September 2014 only and compared the results to when the whole dataset was used.

Finally, as with all assays, the measurement of viremia (viral load) is not without uncertainty. It has been estimated that the standard deviation of the assay is around 0.48 *C*_t_ [15]. To explore the impact of assay uncertainty on our results, we randomly added measurement uncertainty to each viremia measurement and recalculated the relationship between viremia and probability of death over repeated simulations. Details of the simulation study can be found in Section 6 of S1 Appendix. An aggregated dataset is available in S1 Dataset.

### Use of Viremia in Treatment Studies

We compared the number of patients to include in an RCT for a new treatment against Ebola depending on whether viremia was/was not used for stratification of the analysis. We used the Cochran-Mantel-Haenszel test for the stratified analysis and the Chi-squared test for the unstratified analysis. We computed sample sizes using the viremia-level groups identified in this study, with their corresponding CFRs. Sample size formulas are reported in Section 7 of S1 Appendix.

## Results

**Fig 2:**
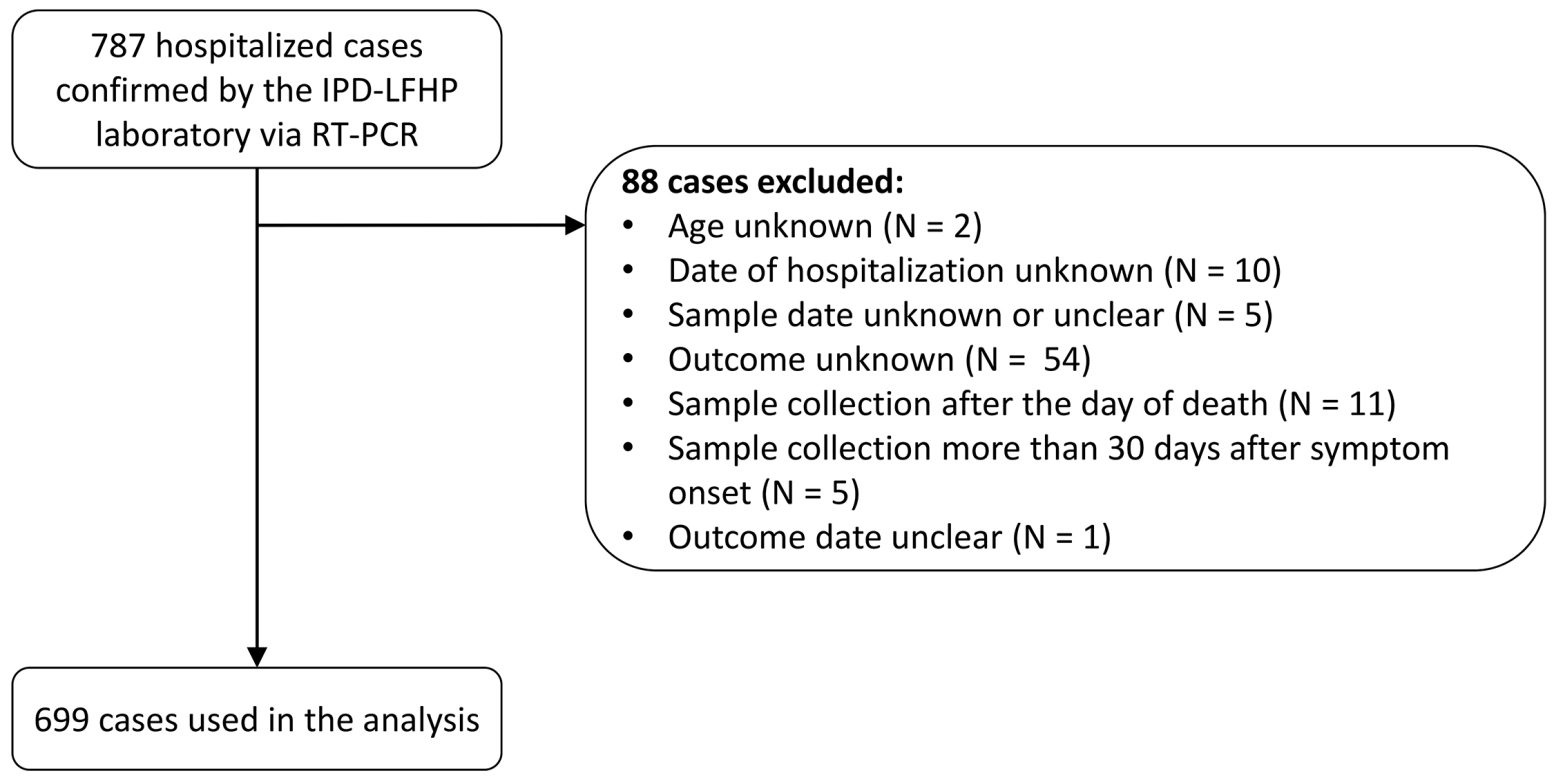
STROBE figure of patients included in this study.

Between 1 March 2014 and 28 February 2015, a total of 1,209 confirmed and probable cases were reported in the study area. Of the 885 (73%) confirmed and probable cases that were hospitalized, 855 (97%) were confirmed, of which 787 (89%) were confirmed by RT-PCR by the IPD-LFHP laboratory and had a viremia measurement available (Fig 2). Eighty-eight cases were excluded from the analysis because of missing or unclear information (*n* = 71), sample collection after death (*n* = 11), or long delays from symptom onset to sample collection/death (*n* = 6) (Fig 2). Our final dataset therefore consisted of 699 cases. Their mean age was 31 y (interquartile range [IQR] 20–42 y), and 47% (*n* = 332) were female (Table 1). Mean times from symptom onset to hospitalization, sample collection, and death were 4.8 (IQR 2–6), 5.6 (IQR 3–7), and 9.3 (IQR 6.5–11.0) d, respectively (Table 1).

**Table 1:** Characteristics of patients included in the study.

Characteristic	Value
Number of patients	699
Age (years), mean (IQR)	31.3 (20.0–42.0)
Female, *n* (proportion)	332 (0.47)
Dead, *n* (proportion)	332 (0.47)
Time from symptom onset to hospitalization (days), mean (IQR)	4.8 (2.0–6.0)
Time from symptom onset to sample collection (days), mean (IQR)	5.6 (3.0–7.0)
Time from symptom onset to death for those who died (days), mean (IQR)	9.3 (6.5–11.0)

**Fig 3:**
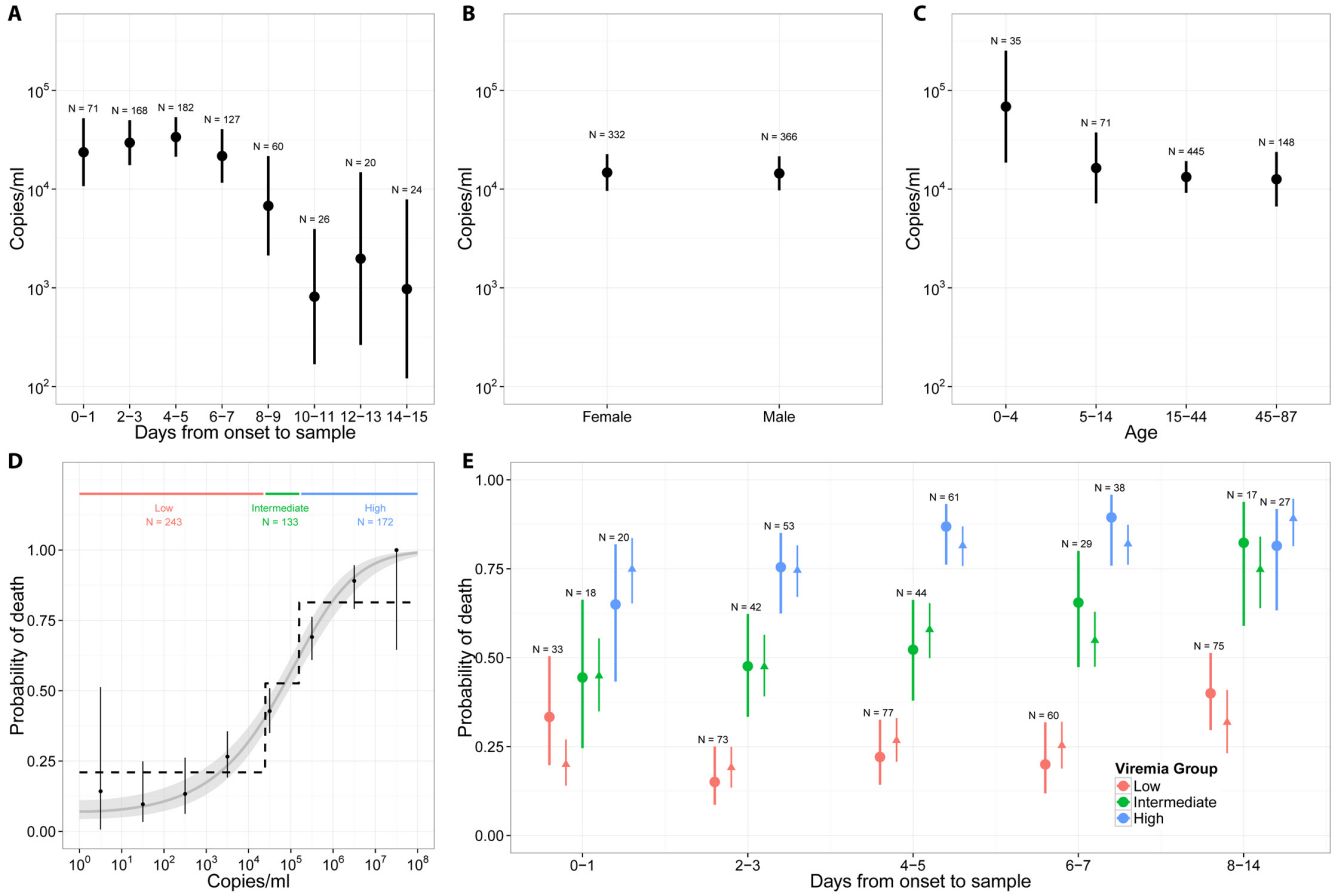
Viremia and the probability of death. (A) Mean viremia as a function of the time from symptom onset to sample collection. (B) Mean viremia by gender. (C) Mean viremia by age group. (D) Probability of death as a function of viremia, when viremia was measured in the week following symptom onset. Three viremia groups are defined: low (*V* < 10^4.4^ copies/ml), intermediate (10^4.4^ ≤ *V* < 10^5.2^ copies/ml), and high (*V* ≥ 10^5.2^ copies/ml) viremia. The probability of death according to viremia group is represented as dotted line. The grey line corresponds to the predictions of the univariable logistic regression model. (E) Probability of death (dot: observed mean; thick line: 95% CI) as a function of the time from symptom onset to sample collection and the viremia group. Mean predicted values obtained with the multivariable logistic regression (triangle) and the bootstrap prediction intervals (thin lines) are also provided.

Fig 3A presents mean viremia as a function of time from symptom onset to sample collection. From day 0 to day 7 after symptom onset, mean viremia was roughly constant, with values around 10^4.45^ (95% CI 10^4.32^–10^4.57^), and it generally declined sharply among patients still alive after day 7. Mean viremia was comparable in males and females (Fig 3B; *p* = 0.95). It was significantly higher in young children (10^4.84^; 95% CI 10^4.27^–10^5.40^) than in other age groups (10^4.13^; 95% CI 10^4.00^–10^4.26^; *p* = 0.02) (Fig 3C).

We restricted the univariable analysis of viremia and outcome to the 548 (78%) patients whose samples were collected within 7 d of symptom onset, a time period when viremia appeared to be stable (Fig 3A). Among these patients, there were a total of 261 deaths (48%). The probability of death was best explained by a logistic regression model with (log_10_ *V*)^2^ as a single explanatory variable (see Section 3 of S1 Appendix). The odds ratio (OR) for a unit increase in (log_10_ *V*)^2^ was 1.12 (95% CI 1.10–1.14). This simple model provided an excellent fit to the data (Fig 3D). The probability of death increased with viremia, from 21% (95% CI 16%–27%; 51 deaths out of 243 individuals) in patients with low viremia (*V* < 10^4.4^) to 53% (95% CI 44%–61%; 70 deaths out of 133 individuals) in those with intermediate viremia (10^4.4^ ≤ *V* < 10^5.2^) and 81% (95% CI 75%–87%; 140 deaths out of 172 individuals) in those with high viremia (*V* ≥ 10^5.2^) (Fig 3D). The proportion of patients in the low, intermediate, and high viremia groups was 44% (95% CI 40%–49%; *n* = 243), 24% (95% CI 21%–28%; *n* = 133), and 31% (95% CI 28%–35%; *n* = 172), respectively. Our results were found to be robust to the level of measurement error expected from the viral load assay (see Section 6 in S1 Appendix).

We performed a multivariable analysis incorporating (log_10_ *V*)^2^, age, and time from symptom onset to sample collection using data from all 699 EVD patients. We found that the OR for a unit increase in (log_10_ *V*)^2^ was unchanged (1.12; 95% CI 1.10–1.14). We also found that late sample collection (>7 d) (OR: 3.04; 95% CI 1.78–5.20) was associated with a significantly increased probability of death compared to those tested <4 d after symptom onset (Table 2). Compared to adults, the probability of death was significantly higher in young children (OR: 2.44; 95% CI 1.02–5.86) and older adults (OR: 2.84; 95% CI 1.81–4.46) and significantly lower in children (OR: 0.46; 95% CI 0.24–0.86) (Table 2), which is consistent with a previous study [10]. This multivariable logistic regression model successfully explained variations in the probability of death by time from symptom onset to sample collection and by viremia group (Fig 3E).

**Table 2:** Odds ratios for death in a multivariate logistic regression performed on all of the 699 cases included in the study.

Variable	Estimated OR (95% CI)	*p*-Value
**(log**_**10**_ **viremia)** ^**2**^	1.12 (1.10–1.14)	<0.001
**Time from symptom onset to sample collection**		
<4 d	Reference	—
4–7 d	1.52 (1.01–2.29)	0.043
>7 d	3.04 (1.78–5.20)	<0.001
**Age group**		
Young children (0–4 y)	2.44 (1.02–5.86)	0.046
Children (5–14 y)	0.46 (0.24–0.86)	0.016
Adults (15–44 y)	Reference	—
Older adults (≥45 y)	2.84 (1.81–4.46)	<0.001

**Fig 4:**
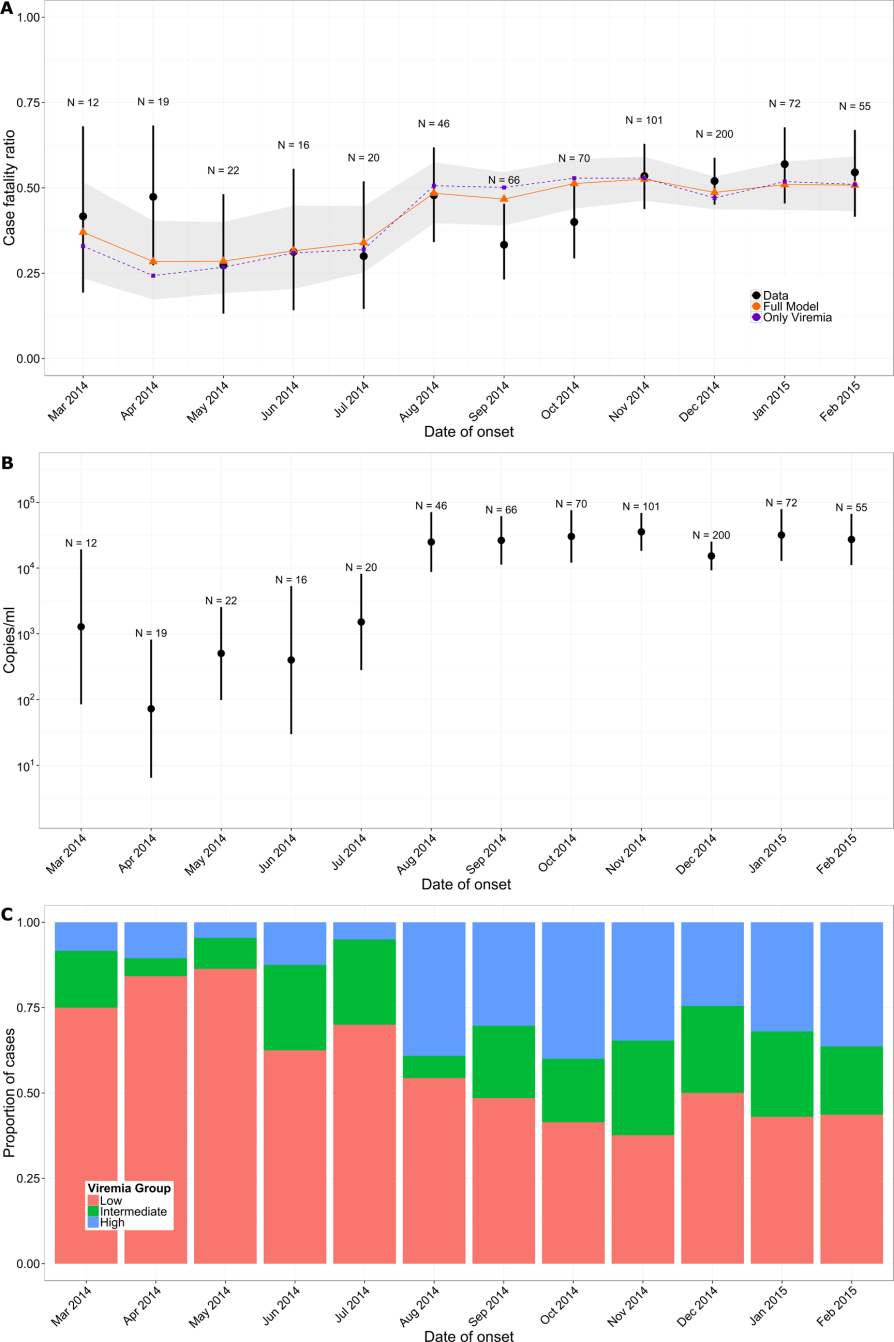
Variation of CFR and viremia over time. (A) Observed CFR by month (black) and predictions obtained from multivariable logistic regression (orange) and from the simple univariable logistic regression model that relies only on viremia (violet). Lines provide 95% CI. The shaded area indicates the bootstrap prediction interval. (B) Mean viremia by month. (C) Proportion of patients in the low (red; *V* < 10^4.4^ copies/ml), intermediate (green; 10^4.4^ ≤ *V* < 10^5.2^ copies/ml), and high (blue; *V* ≥ 10^5.2^ copies/ml) viremia groups by month.

In the study population, the CFR increased from 35% (95% CI 26%–45%) in March–July 2014 to 49% (95% CI 45%–53%) after August 2014 (Fig 4A) (*p* = 0.014). This rise coincided with an order of magnitude increase in mean viremia (Fig 4B) and a surge in the proportion of patients in the high viremia group (Fig 4C) (change in mean viremia in March–July versus after August, *p* < 0.01). Our multivariable logistic regression model performed well in predicting individual outcomes (area under the curve [AUC] of 0.81; see Section 5 of S1 Appendix), and it showed a similar trend in CFR (Fig 4A). A simpler univariable logistic regression model that relied only on viremia showed similar performance (Fig 4A). Finally, a model informed using data from March to September 2014 only (29% of all the data) performed similarly to one trained on the entire dataset (Section 5 of S1 Appendix).

As Ebola viremia is a strong predictor of mortality, comparing patient outcomes within the same viremia category (stratified comparison) will be the most efficient for testing the efficacy of a new treatment. With the mix of viremia levels across patients observed here (low: 44%, intermediate: 24%, high: 31%) and corresponding CFRs, the total number of patients to include in an RCT would be reduced by 25% with a stratified comparison relative to an unstratified comparison (see Section 7 of S1 Appendix). For example, to detect a 20% reduction in CFR as a treatment effect, an RCT would need 438 patients (or 68 for a 50% reduction), while a stratified RCT would need only 324 patients (or 52 for a 50% reduction).

## Discussion

Here, we analyzed a large dataset of laboratory results for 699 EVD patients in order to characterize how the probability of death changed with viremia, while adjusting for age and time between symptom onset and sample collection. We found that viremia was a strong predictor of outcome for individual EVD patients, with the probability of death increasing from 21% in those with low viremia to 81% in those with high viremia. This is important because it confirms the significance of viremia as a predictor of EVD outcome suggested in small case series [16,17] and it provides an important potential measure of risk adjustment in clinical evaluations of Ebola-specific treatments. Important variations in the CFR of the study population (namely, a 14% increase from the first to the second epidemic wave) coincided with an order of magnitude increase in mean viremia (Fig 4B). This general trend for increasing CFR was well captured by a model that adjusted for viremia (Fig 4A).

This finding suggests that heterogeneity in historical CFR estimates among patients, ETCs, and over time may at least partly be explained by variations in viremia and underscores that more valid estimates of the influence of other factors, including treatment effects, might be obtained by adjusting for differing levels of viremia among patients. This finding is particularly important for observational studies that aim to assess the efficacy of treatments as it shows that adjusting for viremia level should reduce confounding. We also provided CFRs for the different viremia groups that could be used as baselines in historical comparisons. Although adjusting for viremia may reduce biases due to an important confounder, it will not account for biases due to other confounders, and the strength of evidence from nonrandomized treatment studies will always be more limited than that obtained with an RCT design.

We showed that analyses stratified by viremia could lead to a 25% reduction in the sample size requirements of RCTs. This calculation was performed under the simple assumption that treatment would have the same impact in the different viremia groups. In practice, this may not be the case, and it will be particularly interesting to assess how efficacy may vary with viremia. It would be straightforward to calculate sample size requirements under other scenarios in which treatment efficacy could be a function of viremia.

We presented estimates of CFR for EVD patients who were hospitalized in the region of Conakry and who had an RT-PCR positive blood sample collected before their date of death. This subset of EVD patients seems a natural candidate to become a historical control group in the context of ongoing treatment studies. However, it should be clear that the CFR in this subset of patients is different (and generally lower) than that of typical probable and confirmed EVD cases. EVD cases that died before they reached the hospital or before a sample could be collected or that were not hospitalized were indeed excluded from our study. To put our estimates in the wider context of the ongoing epidemic, we provide estimates of CFRs for other groups. From the line list, we estimate that the CFR for all probable and confirmed cases with symptom onset between 26 December 2013 and 3 March 2015 in Guinea was 70% (95% CI 68%–72%), consistent with previously reported figures [1,12]. However, it was lower in the Conakry area (64%; 95% CI 61%–67%) than in the rest of the country (74%; 95% CI 72%–75%). In particular, the CFR for hospitalized cases was 51% (95% CI 48%–54%) in the Conakry area compared to 64% (95% CI 61%–66%) elsewhere. Potential explanations for these differences include differences in patient characteristics, time to presentation, capacity for admission to ETCs, severity of EVD in patients who reached ETCs, and/or the early aggressive supportive care treatment approach in the Conakry area from the earliest part of the outbreak [18]. Different eligibility criteria could have been applied to select the final set of patients to be included in the study. In a sensitivity analysis presented in Section 4 of S1 Appendix, we show that the results of our univariable and multivariable analyses were robust to this choice.

The increase in mean viremia and CFR in the study population in the months after July 2014 compared to those before is an interesting observation that requires careful consideration. A number of mechanisms could explain this trend. A first possibility is that disease severity increased during this epidemic. This could have occurred, for example, if the virus evolved to become more virulent. However, such rapid adaptive evolution is not supported by the analysis of the currently available genetic sequences from the outbreak [19,20], although functional analyses would be required to definitely rule out this possibility. Further, it seems unlikely that any increased virulence would be restricted to the Conakry area (where we observed an increase in CFR) and not observed across the whole country (where CFR stayed stable). A second more plausible scenario is that disease severity remained stable over time, but that the ability to detect and hospitalize patients with different levels of disease severity (probability of death) changed. The surge in mean viremia could indicate increased difficulties in detecting and hospitalizing patients with less severe EVD, for example because of resistance from local populations to going to the ETC unless they have very severe illness. It could also be that later in the outbreak, response teams were more successful at detecting severe cases before they died or that the increased number of cases during the peak of the outbreak favored the detection of severe cases. Keeping in mind that we considered only patients who provided a blood sample, a last possibility is that the propensity to perform RT-PCR tests even in EVD patients who were just about to die increased over time. However, in the Conakry area, testing was done systematically when cases arrived to the ETC. Although we cannot be definitive on the mechanism explaining the observed rise in mean viremia, our analysis suggests that, if testing protocols and the virus remain stable over time, monitoring viremia might inform the ability of surveillance systems to detect different levels of disease severity and might be used to compare surveillance systems.

This study has some limitations. Our analysis was conducted on a retrospective cohort using data collected as part of routine case management, with no active follow-up of patients who were discharged. We considered individuals discharged from hospital as having recovered; however, it is possible that some died at a later date. This would result in increases in the CFRs reported here, although any differences are likely to be minor. Further, we used data only on hospitalized cases. Our results may not be generalizable to community cases, as the distribution of viremia may be different in these individuals, for example if they represent cases that died before they could seek care or that had only minor symptoms. The route of infection, which is unknown in our cases, may be linked with differential mortality risk [21]. If such differences exist and are not accompanied by changes in viremia, this could bias our results.

In summary, in a very large and consecutive sample of patients with confirmed EVD, we have shown that viremia is a strong predictor of death that may in part explain previously observed heterogeneity in CFR estimates. Viremia may also provide an important mechanism for risk adjustment among patients in studies aiming to estimate associations of treatment with outcome, and a mechanism to stratify patients into different risk groups within clinical trials.

## Supporting Information

S1 STROBE Checklist (DOC)

S1 Appendix Technical details, further results, and sensitivity analyses. (DOCX)

S1 Dataset Summary dataset. Column 1 gives the number of EVD patients with a specific profile, where the profile is given by the month of hospitalization (column 2), outcome (column 3), age (column 4), and viremia group (column 5).
(CSV)

## Abbreviations

AUC: area under the curve
CFR: case fatality ratio
ETC: Ebola treatment center
EVD: Ebola virus disease
IPD: Institut Pasteur de Dakar
IQR: interquartile range
LFHP: Laboratoire des Fièvres Hémorragiques de Guinée
OR: odds ratio
y.o.: years old
RCT: randomized controlled trial
RT-PCR: reverse transcription PCR
