# Supplementary material for: Use of Viremia to Evaluate the Baseline Case Fatality Ratio of Ebola Virus Disease and Inform Treatment Studies: A Retrospective Cohort Study
Source: PLoS Med. 2015 Dec 1;12(12):e1001908. doi: 10.1371/journal.pmed.1001908 (PMC4666644; doi:10.1371/journal.pmed.1001908)
Supplement: S1 STROBE Checklist — (DOC) [file pmed.1001908.s001.doc]

STROBE Statement—checklist of items that should be included in reports of observational studies

|  | Item No | Recommendation |
| --- | --- | --- |
| **Title and abstract** | 1 | *(*a) Indicate the study’s design with a commonly used term in the title or the abstract  The study’s design is indicated in the subtitle: “a retrospective cohort study”. |
|  |  | (*b*) Provide in the abstract an informative and balanced summary of what was done and what was found  The abstract contains the sections Methods and Findings and Conclusion, where we summarize the results of the paper. |
| Introduction | | |
| Background/rationale | 2 | Explain the scientific background and rationale for the investigation being reported  The first three paragraphs of the Introduction provide the scientific background and rationale for the investigation. |
| Objectives | 3 | State specific objectives, including any prespecified hypotheses  At the end of the Introduction we state that: “We characterize the relationship between a patient’s viremia and their probability of death and demonstrate how this information can be used to explain population-level temporal trends in reported CFRs. Further, we explore how partitioning individuals according to their viremia may inform different treatment study designs.” |
| Methods | | |
| Study design | 4 | Present key elements of study design early in the paper  This is done in the Methods section. |
| Setting | 5 | Describe the setting, locations, and relevant dates, including periods of recruitment, exposure, follow-up, and data collection  This is addressed in subsections “Laboratory work”, “Epidemiological data”, and “Inclusion in the study”. |
| Participants | 6 | *(a) Cohort study*—Give the eligibility criteria, and the sources and methods of selection of participants. Describe methods of follow-up  Eligibility criteria as well as sources and methods of selection are discussed in subsection “Inclusion in the study”. There was no active follow-up of individuals discharged from hospital.  *Case-control study*—Give the eligibility criteria, and the sources and methods of case ascertainment and control selection. Give the rationale for the choice of cases and controls  *Cross-sectional study*—Give the eligibility criteria, and the sources and methods of selection of participants |
|  |  | (*b*) *Cohort study*—For matched studies, give matching criteria and number of exposed and unexposed  NA  *Case-control study*—For matched studies, give matching criteria and the number of controls per case |
| Variables | 7 | Clearly define all outcomes, exposures, predictors, potential confounders, and effect modifiers. Give diagnostic criteria, if applicable  In section “Statistical analysis” we explain that the outcome (survival/death) was modeled using: 1) viremia; 2) age; and 3) time from onset to sample collection.  Confounding was controlled by using a multivariable logistic regression model. |
| Data sources/ measurement | 8* | For each variable of interest, give sources of data and details of methods of assessment (measurement). Describe comparability of assessment methods if there is more than one group  Data collection concerning viral load is described in section “Laboratory work”. For the epidemiological data we point to reference 1. |
| Bias | 9 | Describe any efforts to address potential sources of bias  In the Supplementary Materials we report a sensitivity analysis showing that our results are robust to the choice of the population for analysis (section 3), and we also address the effect of measurement errors (section 5). |
| Study size | 10 | Explain how the study size was arrived at  This is done in the first paragraph of the Results section and in figure 2. |
| Quantitative variables | 11 | Explain how quantitative variables were handled in the analyses. If applicable, describe which groupings were chosen and why  We describe the grouping used for viremia in the “Statistical analysis” section: “We also discretized V into three groups (low: V < v_1_, intermediate: v_1_ ≤ V < v_2_ and high: V ≥ v_2_) and selected the best threshold values v_1_ and v_2_ (v_1_<v_2_) at maximum likelihood (see Supplementary Materials for more details).”  Groupings for age and time from onset to sample collection are described just after the above paragraph: “…: age in 4 classes (infants: 0-4 year old (y.o.), children: 5-14 y.o., adults: 15-44 y.o., older adults: ≥45 y.o.) and time from onset to sample collection in 3 classes (0-3, 4-7, >7 days).” |
| Statistical methods | 12 | *(*a) Describe all statistical methods, including those used to control for confounding  Statistical methods are described in the “Statistical analysis” section. Additional details are also discussed in the Supplementary Materials.  Confounding was controlled by using a multivariable logistic regression model. |
|  |  | (*b*) Describe any methods used to examine subgroups and interactions  NA |
|  |  | (*c*) Explain how missing data were addressed  In section “Inclusion in the study” we specify that cases missing relevant data records were excluded from the analysis: “Case patients were excluded from the analysis if: i) sample collection was done after the day of death or more than 30 days after onset; ii) one of the following variable was missing or unclear: age, prefecture of residence, date of symptom onset, outcome, date of hospitalization, date of death if died, date of sample collection.” |
|  |  | (*d*) *Cohort study*—If applicable, explain how loss to follow-up was addressed  NA  *Case-control study*—If applicable, explain how matching of cases and controls was addressed  *Cross-sectional study*—If applicable, describe analytical methods taking account of sampling strategy |
|  |  | (*e*) Describe any sensitivity analyses  Sensitivity analyses are reported in the Supplementary Materials (section 3). |

Continued on next page

| Results | | |
| --- | --- | --- |
| Participants | 13* | (a) Report numbers of individuals at each stage of study—eg numbers potentially eligible, examined for eligibility, confirmed eligible, included in the study, completing follow-up, and analysed  This is done in the first paragraph of the Results section and in figure 2 (see point c below). |
|  |  | (b) Give reasons for non-participation at each stage  NA |
|  |  | (c) Consider use of a flow diagram  Figure 2 contains the flow diagram. |
| Descriptive data | 14* | (a) Give characteristics of study participants (eg demographic, clinical, social) and information on exposures and potential confounders  Table 1 contains a summary of the characteristics of the patients included in the study. |
|  |  | (b) Indicate number of participants with missing data for each variable of interest  See point 12c. |
|  |  | (c) *Cohort study*—Summarise follow-up time (eg, average and total amount)  NA, see point 6a. |
| Outcome data | 15* | *Cohort study*—Report numbers of outcome events or summary measures over time  In the Results section, we report both numbers and proportion of deaths. The same is done in Table S3 of the Supplementary Materials. |
|  |  | *Case-control study—*Report numbers in each exposure category, or summary measures of exposure |
|  |  | *Cross-sectional study—*Report numbers of outcome events or summary measures |
| Main results | 16 | *(*a) Give unadjusted estimates and, if applicable, confounder-adjusted estimates and their precision (eg, 95% confidence interval). Make clear which confounders were adjusted for and why they were included  We give 95% confidence intervals for all our estimates. |
|  |  | (*b*) Report category boundaries when continuous variables were categorized  See point 11. |
|  |  | (*c*) If relevant, consider translating estimates of relative risk into absolute risk for a meaningful time period  NA |
| Other analyses | 17 | Report other analyses done—eg analyses of subgroups and interactions, and sensitivity analyses  Sensitivity analyses and additional studies on the performance of our models were reported in the Supplementary Materials. |
| Discussion | | |
| Key results | 18 | Summarise key results with reference to study objectives  We begin the Discussion section with a short summary of the main findings of our study. |
| Limitations | 19 | Discuss limitations of the study, taking into account sources of potential bias or imprecision. Discuss both direction and magnitude of any potential bias  In the Discussion section we state: “This study has some limitations. We considered Individuals discharged from hospital as having recovered, however, it is possible that some died at a later date. This would result in increases in the case fatality rates reported here, although any differences are likely to be minor. Further, we only used data on hospitalized cases. Our results may not be generalizable to community cases as the distribution of viremia may be different in these individuals, for example if they represent cases that died quickly before they could seek care or only had minor symptoms. The route of inoculation, which is unknown in our cases, may be linked with differential mortality risk. If such differences do exist and they are not accompanied by similar changes in viral load, this could bias our results.” |
| Interpretation | 20 | Give a cautious overall interpretation of results considering objectives, limitations, multiplicity of analyses, results from similar studies, and other relevant evidence  At the end of the Discussion section we clearly state the limitations of our study (see point 19). |
| Generalisability | 21 | Discuss the generalisability (external validity) of the study results  In the Conclusion we state that “we have shown that viremia is a strong predictor of death that may in part explain previously observed heterogeneity in CFR estimates and may also provide an important mechanism to both risk adjust among patients in studies aiming to estimate associations of treatment with outcome, and a mechanism to stratify patients into different risk groups within clinical trials.” |
| Other information | | |
| Funding | 22 | Give the source of funding and the role of the funders for the present study and, if applicable, for the original study on which the present article is based  Funding sources are acknowledged at the end of the paper. |

*Give information separately for cases and controls in case-control studies and, if applicable, for exposed and unexposed groups in cohort and cross-sectional studies.

**Note:** An Explanation and Elaboration article discusses each checklist item and gives methodological background and published examples of transparent reporting. The STROBE checklist is best used in conjunction with this article (freely available on the Web sites of PLoS Medicine at http://www.plosmedicine.org/, Annals of Internal Medicine at http://www.annals.org/, and Epidemiology at http://www.epidem.com/). Information on the STROBE Initiative is available at www.strobe-statement.org.
