# Supplementary material for: Use of Viremia to Evaluate the Baseline Case Fatality Ratio of Ebola Virus Disease and Inform Treatment Studies: A Retrospective Cohort Study
Source: PLoS Med. 2015 Dec 1;12(12):e1001908. doi: 10.1371/journal.pmed.1001908 (PMC4666644; doi:10.1371/journal.pmed.1001908)
Supplement: S1 Appendix — (DOCX) [file pmed.1001908.s002.docx]

**Supporting Information**

**Use of Viremia to Evaluate the Baseline Case Fatality Ratio of
Ebola Virus Disease and Inform Treatment Studies: A Retrospective Cohort Study**

**Contents**

[1 Timeline of analysis plans 2](#_Toc433971803)

[2 Defining viremia groups via maximum likelihood clustering 2](#_Toc433971804)

[3 Logistic regression model selection 2](#_Toc433971805)

[4 Sensitivity analysis 3](#_Toc433971806)

[4.1 All cases versus cases with a sample collected within 7 days of onset 3](#_Toc433971807)

[4.2 Varying eligibility criteria 5](#_Toc433971808)

[5 Model Performance 8](#_Toc433971809)

[6 Effect of measurement errors 9](#_Toc433971810)

[7 Stratification of RCTs by viremia 10](#_Toc433971811)

[8 References 11](#_Toc433971812)

# Timeline of analysis plans

In November 2014, SC, HS and AAS discussed the possibility to run explanatory analyses on data collected as part of routine EVD case management in Conakry with a view to describe viremia variations in EVD patients, ascertain factors that could explain these variations and evaluate whether viremia could be used to predict outcome (this latter hypothesis had been suggested in a prior study with small sample size [1]). In November 2014, HS performed a first simple descriptive analysis from a sample of 83 EVD patients for whom viremia was available. However, the relatively limited sample size prevented a thorough assessment.

In April 2015, we restarted exploring these questions when a larger dataset (presented in this paper) became available. In this second round of analysis, we also decided to explore whether variations of viremia in reported cases might provide insights about variations in detection rates.

# Defining viremia groups via maximum likelihood clustering

We partition EVD cases into k=1…K groups according to their viremia. Denoting *p_k_* the probability of death for an individual belonging to viremia group *k*, the likelihood function can be written as:

$$L=\prod_{k=1\ldots K} {(1-p_{k})}^{N_{k}-D_{k}} p_{k}^{D_{k}}$$

where *N_k_* and *D_k_* are respectively the number of individuals and the number of deaths in viremia group *k*.

In our case, the three clusters - corresponding to the three viremia groups discussed in the main text - are defined via two viremia cut-points: the first representing the threshold between the low and the intermediate viremia groups, and the second representing the threshold between the intermediate and the high viremia groups. Such cut-points are then estimated by maximizing the likelihood function defined above.

# Logistic regression model selection

Model selection was performed using the Akaike information criterion (AIC) to select the logistic regression model that best explained the role of viremia on individual probability of death. Data was restricted to the 548 cases with sample collected within seven days from symptom onset. We tested several polynomial models up to order 5, as set out below. The results, shown in S1 Table, indicate that the best fit corresponds to the model with a single (log_10_ V)^2^ term.

The best polynomial term identified above was then used in the multivariable model.

**S1 Table: Model selection**

| **Explanatory Variable** | **AIC** |
| --- | --- |
| log_10_ V | 605.65 |
| log_10_ V+ (log_10_ V)^2^ | 595.20 |
| **(log_10_ V)^2^** | **594.84** |
| log_10_ V+ (log_10_ V)^2^+ (log_10_ V)^3^ | 596.96 |
| (log_10_ V)^2^+ (log_10_ V)^3^ | 596.30 |
| (log_10_ V)^3^ | 595.14 |
| log_10_ V+ (log_10_ V)^2^+ (log_10_ V)^3^+ (log_10_ V)^4^ | 598.51 |
| log_10_ V+ (log_10_ V)^2^+ (log_10_ V)^3^+ (log_10_ V)^4^+ (log_10_ V)^5^ | 600.50 |

# Sensitivity analysis

Different criteria can be applied to select the final set of patients to be included in the study. In this sensitivity analysis, we show that the results of viremia as a predictor of outcome are robust to the choice of the population for analysis and independent of other predictors.

## All cases versus cases with a sample collected within 7 days of onset

Here, we compare the results of models using all cases and models restricted to individuals who had blood collected within 7 days of symptom onset. We also examine whether age and time of sampling could confuse the association between viremia and CFR.

We find that results of the univariable and multivariable logistic regressions are robust to this choice (S2 Table). Comparing the estimates from the univariable and multivariable analyses shows that the odds-ratio of viremia for death is little affected by the inclusion of age and time from onset to sample collection as adjustment factors. It also shows that restricting the analysis to the cases sampled within 7 days from onset yielded very similar results to the whole data analysis.

**S2 Table: Odds ratios for death in univariable and multivariable logistic regression performed on all 699 cases included in the study and on the 548 cases that had a sample collected within 7 d of symptom onset.**

| **Variable** | **All Cases** | | **Cases with viremia sample within 7 days from onset** | |
| --- | --- | --- | --- | --- |
|  | **(N=699)** | | **(N=548)** | |
|  | **Univariable analysis** | **Multivariable analysis** | **Univariable analysis** | **Multivariable analysis** |
|  | **Odds Ratio (95% CI)** | **Odds Ratio (95% CI)** | **Odds Ratio (95% CI)** | **Odds Ratio (95% CI)** |
| (Log10 Viremia)^2^ | 1.10 (1.09-1.12) | 1.12 (1.10-1.14) | 1.12 (1.10-1.14) | 1.13 (1.10-1.15) |
| Delay from onset to sample collection |  |  |  |  |
| *< 4 days* | - | 1 | - | 1 |
| *4-7 days* | - | 1.52 (1.01-2.29) | - | 1.54 (1.02-2.33) |
| *> 7 days* | - | 3.04 (1.78-5.20) | - | - |
| Age group |  |  |  |  |
| *Infant (0-4)* | - | 2.44 (1.02-5.86) | - | 3.20 (1.19-8.64) |
| *Children (5-14)* | - | 0.46 (0.24-0.86) | - | 0.45 (0.22-0.92) |
| *Adults (15-44)* | - | 1 | - | 1 |
| *Older Adults (45-87)* | - | 2.84 (1.81-4.46) | - | 2.98 (1.76-5.06) |

## Varying eligibility criteria

Our baseline analysis presented in the main manuscript excluded cases that had a sample collected after the date of death (scenario D_1_). Here we explored how results were modified when the following alternative selection criteria were considered:

- **Subset D_0_ (N=710):** EVD patients from the linelist that i) were confirmed by a RT-PCR test performed by the Institut Pasteur de Dakar laboratory in Conakry, ii) resided in Conakry or in the surrounding prefectures of Boffa, Coyah, Dubreka, Forecariah, Fria, Kindia and Telimele (in which diagnoses were mostly performed by our laboratory), iii) had symptom onset between 1 March 2014 and 28 February 2015, iv) were hospitalized, v) died within 50 days from onset (if dead), vi) had known age, prefecture of residence, date of symptom onset, outcome, date of hospitalization, date of death if died, date of sample collection.
- **Subset D_1_ (baseline used in the main text) (N=699):** all cases from D_0_ that had a sample collected before or on the day of death (if dead).
- **Subset D_2_ (N=651)**: all cases from D_0_ that had a sample collected strictly before the day of death (if dead).
- **Subset D_3_ (N=550)**: all cases from D_0_ that had a sample collected before or on the day of hospitalization.
- **Subset D_4_ (N=622)**: all cases from D_0_ that had a sample collected up to 1 day after hospitalization.
- **Subset D_5_ (N=640)**: all cases from D_0_ that had a sample collected up to 2 days after hospitalization.

S3 Table shows how results of the univariable analyses presented in Figure 3D would be affected by a change of selection criteria. As in Figure 3D, this analysis is restricted to patients with a sample collected within 7 days of onset.

S4 Table presents results of the sensitivity analysis for the multivariable logistic regression presented in Table 2.

Results of both the univariable and multivariable analyses are robust to the selection criteria.

**S3 Table: Sensitivity analysis for the univariable analyses using viremia measured within 7 d of symptom onset to predict death. We explore how results are affected by changes in case selection criteria. Results for our baseline scenario are presented in Fig. 3D.**

|  | **Subset D_0_** | **Baseline subset D_1_** | **Subset D_2_** | **Subset D_3_** | **Subset D_4_** | **Subset D_5_** |
| --- | --- | --- | --- | --- | --- | --- |
| *N* | 555 | 548 | 511 | 461 | 518 | 532 |
|  |  |  |  |  |  |  |
| ***Univariable logistic regression*** |  |  |  |  |  |  |
| Odds Ratio (Log10 Viremia)^2^ | 1.12 (1.10-1.14) | 1.12 (1.10-1.14) | 1.13 (1.11-1.16) | 1.12 (1.10-1.15) | 1.12 (1.10-1.15) | 1.12 (1.10-1.15) |
|  |  |  |  |  |  |  |
| ***Clustering of patients according to viremia*** |  |  |  |  |  |  |
| Low viremia group |  |  |  |  |  |  |
| *N* | 245 | 243 | 228 | 203 | 223 | 231 |
| *Deaths* | 53 | 51 | 36 | 41 | 46 | 47 |
| *Probability of death* | 0.22 (0.17-0.27) | 0.21 (0.16-0.27) | 0.16 (0.12-0.21) | 0.20 (0.15-0.26) | 0.21 (0.16-0.26) | 0.20 (0.16-0.26) |
|  |  |  |  |  |  |  |
| Intermediate viremia group |  |  |  |  |  |  |
| *N* | 134 | 133 | 125 | 211 | 128 | 130 |
| *Deaths* | 71 | 70 | 62 | 135 | 67 | 68 |
| *Probability of death* | 0.53 (0.45-0.61) | 0.53 (0.44-0.61) | 0.50 (0.41-0.58) | 0.64 (0.57-0.70) | 0.52 (0.44-0.61) | 0.52 (0.44-0.61) |
| High viremia group |  |  |  |  |  |  |
| *N* | 176 | 172 | 158 | 47 | 167 | 171 |
| *Deaths* | 144 | 140 | 126 | 45 | 135 | 139 |
| *Probability of death* | 0.82 (0.75-0.87) | 0.81 (0.75-0.87) | 0.80 (0.73-0.85) | 0.96 (0.86-0.99) | 0.81 (0.74-0.86) | 0.81 (0.75-0.86) |

**S4 Table: Sensitivity analysis for the multivariable analysis using viremia, age, and time from symptom onset to sample collection to predict death. We explore how results are affected by changes in case selection criteria. Results for our baseline scenario are presented in Table 2.**

| **Odds Ratio** | **Subset D_0_** | **Baseline subset D_1_** | **Subset D_2_** | **Subset D_3_** | **Subset D_4_** | **Subset D_5_** |
| --- | --- | --- | --- | --- | --- | --- |
| (Log10 Viremia)^2^ | 1.12 (1.10-1.14) | 1.12 (1.10-1.14) | 1.13 (1.11-1.15) | 1.12 (1.09-1.14) | 1.12 (1.09-1.14) | 1.12 (1.10-1.14) |
| Delay onset sample collection |  |  |  |  |  |  |
| *< 4 days* | 1.00 | 1.00 | 1.00 | 1.00 | 1.00 | 1.00 |
| *4-7 days* | 1.49 (0.99-2.24) | 1.52 (1.01-2.29) | 1.26 (0.82-1.93) | 1.17 (0.75-1.81) | 1.30 (0.86-1.96) | 1.33 (0.88-2.00) |
| *> 7 days* | 3.10 (1.83-5.25) | 3.04 (1.78-5.20) | 2.88 (1.64-5.06) | 3.14 (1.67-5.89) | 4.09 (2.28-7.35) | 4.49 (2.51-8.04) |
| Age group |  |  |  |  |  |  |
| *0-4* | 2.31 (0.96-5.54) | 2.44 (1.02-5.86) | 2.28 (0.91-5.69) | 2.66 (1.01-6.96) | 2.70 (1.06-6.88) | 2.72 (1.06-6.98) |
| *5-14* | 0.43 (0.23-0.82) | 0.46 (0.24-0.86) | 0.48 (0.24-0.94) | 0.57 (0.29-1.14) | 0.49 (0.26-0.95) | 0.49 (0.26-0.94) |
| *15-44* | 1.00 | 1.00 | 1.00 | 1.00 | 1.00 | 1.00 |
| *45-87* | 2.68 (1.71-4.20) | 2.84 (1.81-4.46) | 2.83 (1.75-4.56) | 2.95 (1.75-4.98) | 2.66 (1.64-4.33) | 2.87 (1.77-4.64) |

# Model Performance


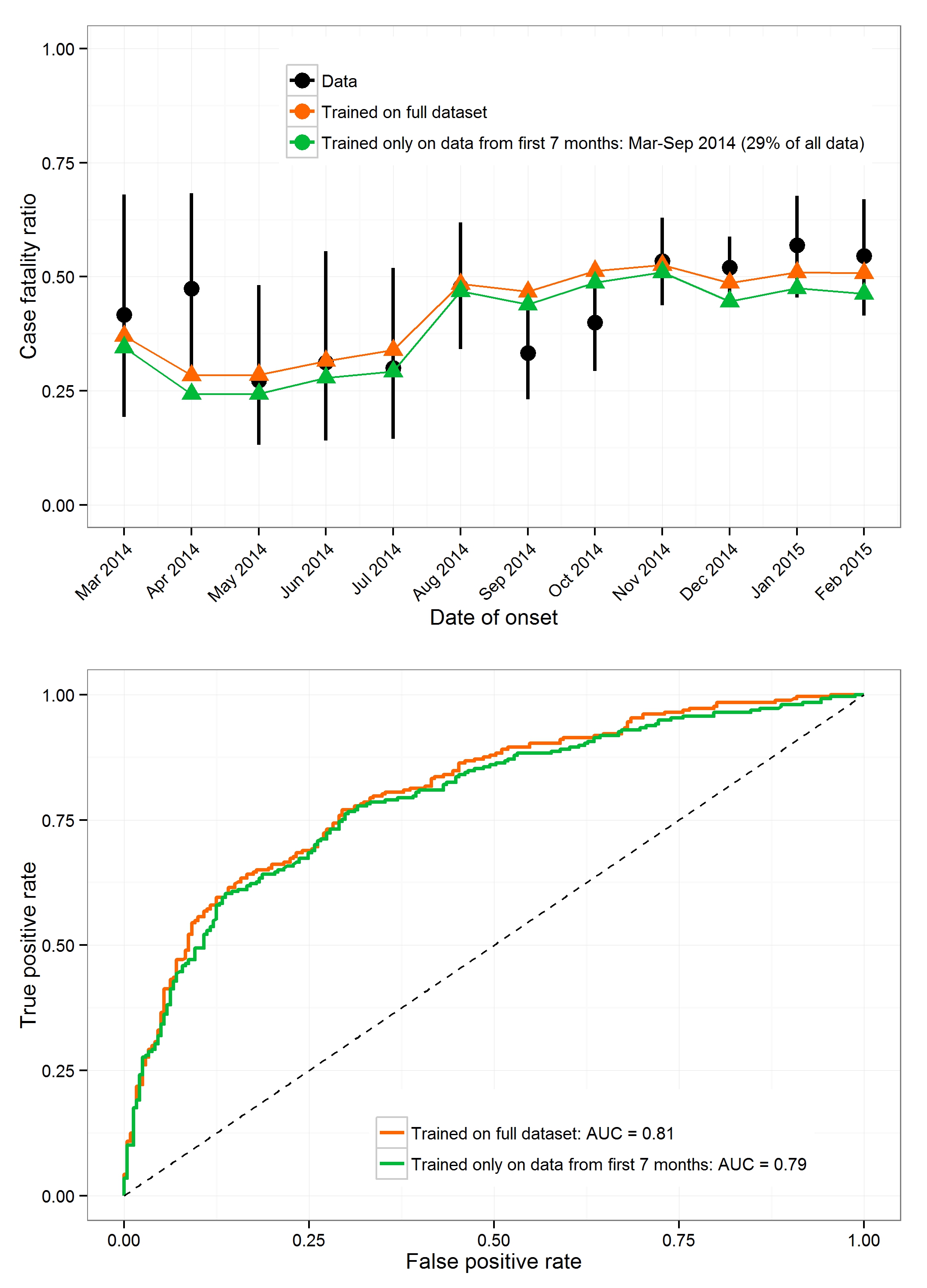


**S1 Fig: Model performance**. **A.** Estimated monthly CFRs obtained with a multivariable logistic regression model that was trained using cases hospitalized between March and September 2014 only (green) and with a model trained using the entire dataset (orange). **B.** Receiver operating characteristic curves for the same classifiers shown in A, with the same color code.

# Effect of measurement errors

To quantify the impact that measurement errors could have on the results reported in the main text, we simulated 1,000 synthetic datasets. These were generated by adding Gaussian noise with a mean of zero and standard deviation of 0.48 CT (following the estimates of measurement uncertainty reported in Weidmann et al., [2]) to the raw CT values obtained with RT-PCR.

For each of these 1,000 synthetic datasets, we performed the univariable analysis (using all cases having sample collected in the week following symptom onset) and the multivariable analyses (on all cases having symptom onset from March to September 2014).

In Panel A of S2 Fig, we plot the median probability of death as a function of viremia derived from the 1,000 univariable analyses with 95% uncertainty range. In Panel B of S2 Fig, we present the distribution of the AUC derived from the 1,000 multivariable analyses. These analyses show that our findings are robust to viral load measurement error.


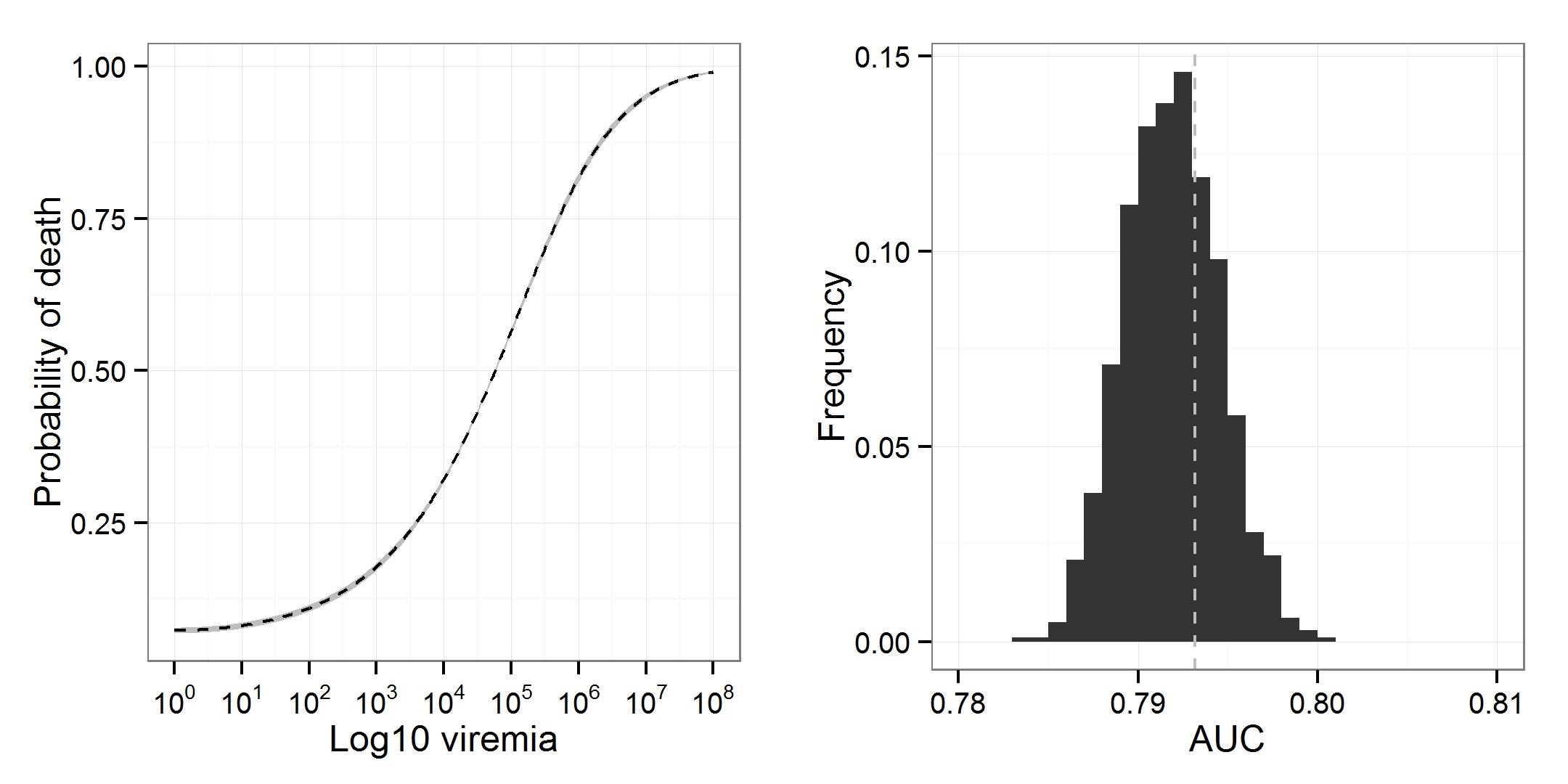


**B**

**A**

**S2 Fig: Effect of measurement error**. **A.** Probability of death as a function of viremia derived from 1,000 synthetic datasets with measurement error. Each synthetic dataset was generated by adding Gaussian noise with mean 0 and standard deviation 0.48 [2] to raw *C_t_* values recorded from cases with blood collected within a week of symptom onset (N=548). For each synthetic dataset we recalculated the coefficient linking (log_10_ V)^2^ and probability of death. The grey area represents the 95% uncertainty range of the resulting distribution. The dashed black line corresponds to the curve for the original dataset. **B.** Histogram of the AUC scores obtained with the multivariable logistic regression for 1,000 synthetic datasets. For each dataset, the model was trained on cases having symptom onset from March to September 2014 and validated on cases with symptom onset from October 2014 to February 2015. The vertical grey line corresponds to the AUC value in the original dataset.

# Stratification of RCTs by viremia

In a RCT for Ebola treatment with two arms, patients would be randomized to either a new treatment or best of care (placebo or control). The number of patients to include in each arm is computed so that the final comparison in outcomes has large power, i.e. a high probability to find evidence of a treatment effect if it exists.

If a baseline patient characteristic is a predictor of the outcome, for example viremia for death, stratifying on this characteristic allows a smaller sample size and improves feasibility of the RCT. With stratification, patients would first be classified according to, say, high/low viremia, and randomization carried out within each category. In this way, the natural variability in outcome between those who receive the new treatment and the others is reduced and fewer patients required for the same power.

Assuming death/recovery as the outcome, the reduction in sample size due to stratification can be computed [2] . With simple randomization, the primary analysis is a chi-squared test on the cross-tabulation of treatment and outcome; whereas with stratified randomization cross-tabulation is done in each viremia category and the Cochran-Mantel-Haenszel test is used.

Assuming *K* categories for the stratified randomization, each with probability *p_i_* and case fatality ratio ${CFR}_{i}^{0}$, we write ${CFR}_{T}^{0}= \sum_{i} p_{i} {CFR}_{i}^{0}$ as the average CFR. We further assume that the new treatment reduces mortality by the same fraction *RR* in each group, i.e. CFR in the treated is ${CFR}_{i}^{1}=RR {CFR}_{i}^{0}$. We also write

$\upsilon_{i}^{z}={CFR}_{i}^{z}\left( 1-{CFR}_{i}^{z} \right); \upsilon_{T}^{z}= \sum_{i} p_{i} \upsilon_{i}^{z}; \psi=\sum_{i<j} p_{i}p_{j}\left( {CFR}_{i}-{CFR}_{j} \right)^{2}$.

The total number of patients *n_random_* to include in a fixed size trial with simple randomization to evidence a treatment effect *RR* with power 1-β is:

$$n_{random}={2\left( \frac{z_{1-\alpha/2}\sqrt{2 \left( \upsilon_{T}^{0}+\psi\right)}+z_{1-\beta}\sqrt{\upsilon_{T}^{0}+\upsilon_{T}^{1}+\psi\left( 1+RR \right)}}{\left( 1-RR \right) {CFR}_{T}^{0}} \right)}^{2}$$

whereas in the stratified design, *n_strata_* patients are required :

$$n_{strata}=2\left( \frac{z_{1-\alpha/2}\sqrt{2 \upsilon_{T}^{0}}+z_{1-\beta}\sqrt{\upsilon_{T}^{0}+\upsilon_{T}^{1}}}{\left( 1-RR \right) {CFR}_{T}^{0}} \right)^{2}$$

Further reduction in the sample size could be obtained using stratified sequential designs.

# References

1. Towner JS, Rollin PE, Bausch DG, Sanchez A, Crary SM, Vincent M, et al. Rapid diagnosis of Ebola hemorrhagic fever by reverse transcription-PCR in an outbreak setting and assessment of patient viral load as a predictor of outcome. J Virol. 2004;78(8):4330-41. doi: Doi 10.1128/Jvi.78.8.4330-4341.2004. PubMed PMID: WOS:000220641600052.

2. Nam J. Sample size determination for case-control studies and the comparison of stratified and unstratified analyses. Biometrics. 1992;48(2):389-95. PubMed PMID: 1637968.
